# A mobile swabbing booth to address Singapore GPs’ concerns about swabber protection: human-centred design during the COVID-19 pandemic

**DOI:** 10.1186/s12875-021-01531-8

**Published:** 2021-09-08

**Authors:** Boon See Teo, Esther Li, Yi-Lin Khoo, Michelle Evaristo, Yang Fang, Helen E. Smith

**Affiliations:** 1 Camry Medical Centre, 95 Toa Payoh Lorong 4 #01-66, Singapore, 310095 Singapore; 2 Lee Kong Chian School of Medicine, Nanyang Technological University, Experimental Medicine Building, 59 Nanyang Drive, Singapore, 636921 Singapore; 3 Yong Loo Lin School of Medicine, National University of Singapore, NUS Yong Loo Lin School of Medicine NUHS Tower Block, 1E Kent Ridge Road Level 11, Singapore, 119228 Singapore; 4 Duke-NUS Medical School, 8 College Road, Singapore, 169857 Singapore; 5 Temasek International, 60B Orchard Road, #06-18 Tower 2, The Atrium@Orchard, Singapore, 238891 Singapore

**Keywords:** Quality improvement, Safety management, Ergonomics, Infection control, Primary Health Care, Pandemic, Swabbing booth, Safety, COVID-19

## Abstract

**Background:**

During the COVID-19 pandemic, the Ministry of Health asked Singapore’s private general practitioners (GPs) to perform swab testing in their clinics, but some GPs had concerns about swabber protection. Our aim was to develop a swabbing booth to address these concerns.

**Methods:**

We developed a prototype with potential GP users using a human-centred design approach and piloted it with 10 GP clinics. The pilot was then extended to 170 GP clinics around Singapore. These GPs were then surveyed on user satisfaction.

**Results:**

Ninety-three GPs (54%) responded. The majority (75%) practiced in public residential estates in small practices (mean 1.95 doctors). 86% requested the booth to enhance swabber protection. 74% “would recommend” or “would strongly recommend” the booth to colleagues. 79% continue to use the booth to conduct swab tests. 92% liked that it offered swabber protection. 71% liked that the booth created a separate space for swabbing and 64% liked its ease of disinfection. 47% started swabbing only after receiving the booth and 58% said the booth was “important” or “very important” to their decision to participate in swab testing. However, 34% disliked that it took up too much space and the most frequently critiqued area was the gloves.

**Conclusion:**

The human-centred design approach generated a product that had high user satisfaction, addressed GPs’ concerns of swabber protection and increased GPs’ participation in swab testing. The booth may be useful where GPs are concerned about swabber protection and space is limited.

**Supplementary Information:**

The online version contains supplementary material available at 10.1186/s12875-021-01531-8.

## Introduction

Private general practitioners (GPs) are in the frontline of Singapore’s response to the COVID-19 pandemic. There are 1700 private GP clinics in Singapore, which provide 80% of Singapore’s primary care [1]. Mostly situated in residential estates, they are the point of first contact in the health system for most patients. About 930 of these clinics are designated Public Health Preparedness Clinics (PHPCs) [2] and they provide subsidised treatment, investigations and medications during public health emergencies [3].

Upper respiratory tract infections make up 44% of GPs’ acute caseloads [4]. Beginning in January 2020, GPs were asked to refer suspected cases of COVID-19 to hospitals for swab testing. Amid rising daily cases in March 2020, the Ministry of Health encouraged PHPCs to participate in the Swab-and-Send Home (SASH) Programme, which aimed to expand disease surveillance and support rapid case finding [5]. PHPCs on SASH would offer patients with acute respiratory illness same-day COVID-19 swab testing within the clinic. However, GPs were concerned about swabber protection and premise contamination as patients often coughed or sneezed while being swabbed. In April 2020, around 140 out of 930 PHPCs had joined the programme [2].

There are few studies about transmission risk during nasopharyngeal or oropharyngeal swabs [6]. However, it is reasonable to assume that the transmission risk to swabber is high, as during swabbing the potentially infectious patient stands less than a metre away, his face is exposed, and he may cough. These three factors multiply the risk to many times that of a typical interaction with masks worn, more than a metre apart and without coughing [7, 8]. Furthermore, as SARS-CoV-2 transmission is via droplets, aerosols and fomites, and transmission risk is greater in confined spaces [9], there may be risks to other users of the space, raising the issue of disinfection.

Infection control guidelines for COVID-19 swab testing generally specify that Personal Protective Equipment (PPE) be worn to protect the healthcare worker [10–13]; however, PPE itself can be soiled or fit poorly. The guidelines specified additional measures such as designating a separate area or room for swabbing [10–12], disinfection of the space [10], a physical barrier between patient and swabber [12] or even patient self-swabbing [11].

Swabbing booths were a potential solution to the problem of infection control. These had been deployed to enhance protection for swabbers in various settings both locally [14] and internationally [15–17], but to our knowledge, none in the GP setting. A swabbing booth is able to designate a separate easy-to-disinfect swabbing area with a physical barrier between patient and healthcare worker. The Temasek Foundation (TF), a Singaporean philanthropic organization, collaborated with a PHPC, Camry Medical Centre (CMC), and a precision engineering company, Applied Total Control Treatment Pte Ltd. (ATC), to design and build a swabbing booth that would address the safety concerns of PHPC GPs.

We followed human-centred design (HCD) principles to develop the prototype. HCD is defined by psychologist Donald Norman as “the process that ensures that the designs match the needs and capabilities of the people for whom they are intended” [18]. It is characterised by iterative cycles of observation of users, idea generation, prototyping and user testing.

Our goal was to design a swabbing booth that addressed the GPs’ concerns about swabber protection and to evaluate user satisfaction.

## Method

The project had 3 phases: design development, piloting and user survey. We started the design development on 14th May 2020 and closed the survey on 24th September 2020.

Design development (Phase 1) involved 4 GP testers (2 male, 2 female) from 3 PHPCs. Discussions and trials involving the GP testers, ATC engineers, and TF volunteers were conducted at a PHPC (CMC). The GPs reviewed preliminary prototypes and defined 6 functional requirements for a booth (see Table 1): swabber protection, ease of disinfection, outdoor use, mobility, good ergonomics and patient privacy.

**Table 1:** Key user requirements and design strategies

GPs’ requirements	Design strategies adopted
**Swabber protection** Swabbers should be protected from droplets produced by coughing and sneezing during swabbing.	• A full-height cubicle served as a barrier between patient and swabber, with a roof to block upward transmission trajectory. • The joints of the structure were sealed to prevent droplet transmission.
**Ease of disinfection** Wipe-down had to be simple as the booth would be disinfected between patients.	• For the panels, polycarbonate was chosen over acrylic as polycarbonate could withstand wipe-downs with alcohol. • Surfaces were made as smooth as possible with no nooks and crannies.
**Outdoor or semi-outdoor use** GPs should be able to place it outside the clinic to segregate swabbing space from consultation space, for infection control.	• Aluminium and polycarbonate were chosen for their weather-resistance. • No electrical components were included. • The cubicles were open, without doors, to allow wind, humidity, heat and sunlight to combat pathogens. This would also reduce the number of surfaces needing wipe-down between patients.
**Mobility** In order to be stored indoors after hours, it had to be sufficiently compact to fit within small clinic spaces, and require minimal manpower to set up as GP clinics run on lean teams.	• Castors and handles were added. • It was made narrow enough to pass through standard doorways. • The footprint was made just large enough to contain both swabber and patient (600x800mm). • Lightweight materials and compact size made it easy for a single clinic staff to move and set up.
**Good ergonomics** It should be comfortable for the swabber to perform the procedure. It should also accommodate patients of different builds.	• Dimensions were specified for a standing swabber performing a nasopharyngeal swab on a patient 1.10–1.75 m tall. Shorter patients could stand on a stool and taller patients could be seated. • Glove ports were fixed at a comfortable height for testers who were 1.55–1.75 m tall. • Gloves had to be touch-sensitive, low cost and easy to replace. • Curved shelves in both cubicles provided space for swabbing equipment to be placed.
**Patient privacy** If the swabbing was done outside the clinic in a public area, patient privacy had to be respected.	• Semi-opaque cubicles for swabber and patient provided some privacy while allowing light to pass through for swabbing.

Subsequently, CMC, TF and ATC developed the design in 3 iterations, each involving:

- Idea generation: CMC translated the GP testers’ inputs into a design drawing.
- Prototyping: ATC built a prototype based on the design drawing.
- Testing: The completed prototypes were tested on-site in CMC. GP testers role-played as swabber and patient, performing pretend swabs to test booth ergonomics, and provided feedback. Patients were not involved in testing at this stage.
- Observation: TF and CMC consolidated the feedback. Design decisions were then translated into a final design drawing. Table 1 summarises the design strategies.

**Fig. 1:**
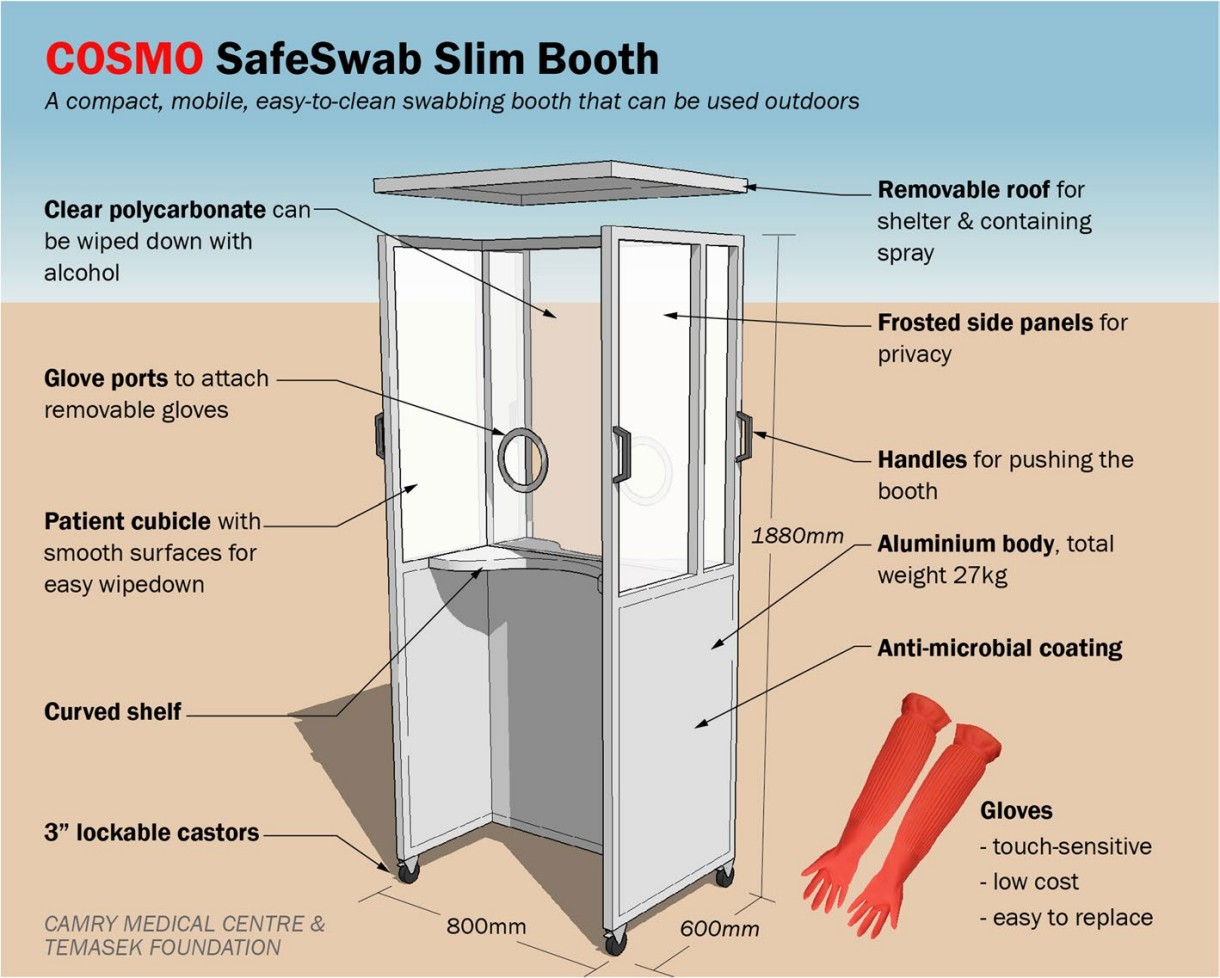
Final design of mobile swabbing booth

**Fig. 2:**
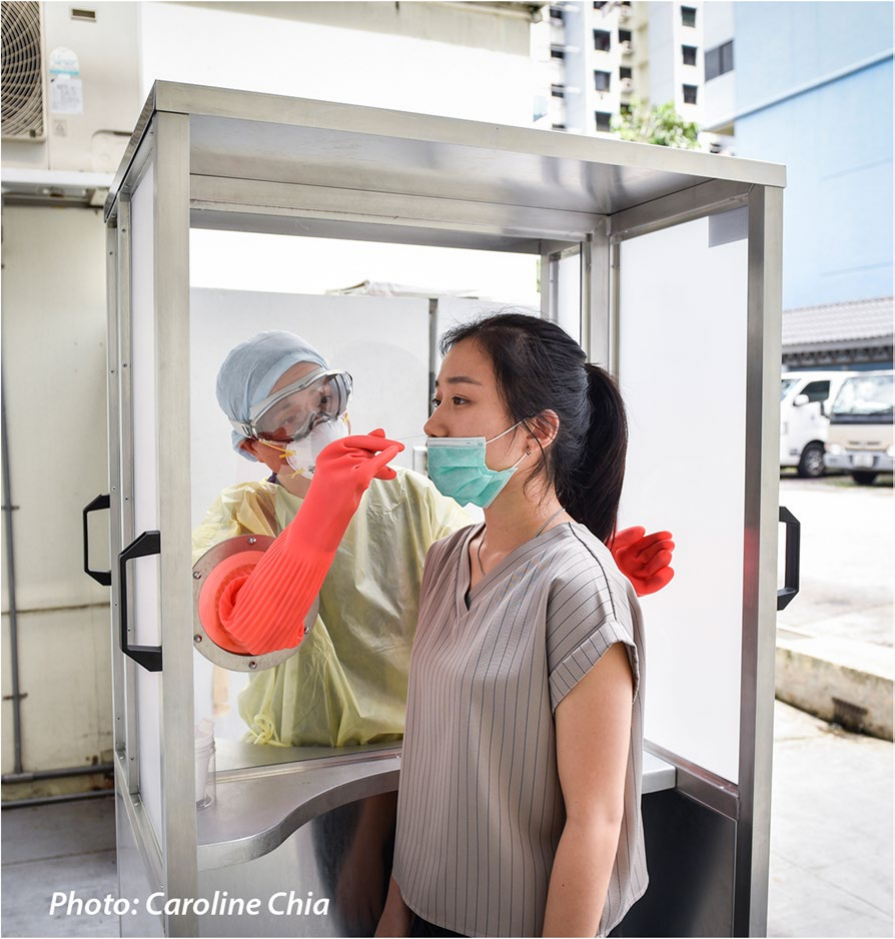
Photograph of the booth

In all, five prototypes of booths and four models of gloves were tested. The final booth design (see Fig. 1 and Fig. 2) had separate cubicles for swabber and patient. It was mobile and slim enough to go through doorways, and light enough to be moved by one person. We chose a model of gloves that fulfilled testers’ requirements for tactile-sensitivity, ease of disinfection, ease of replacement and low cost.

Piloting (Phase 2) was conducted in 10 clinics from different towns in Singapore to test the feasibility of the booth prototype. Over 2 weeks, 10 pilot GP participants used the booth to swab patients and provide feedback via a chat group. The GPs suggested two refinements: to increase the shelf size on the patient side to prevent swab kits from falling off, and to provide larger gloves. The refined prototype was then produced.

We then extended the pilot to all PHPC GPs, inviting applications for a booth via a mass e-mail from the College of Family Physicians to their members, and via messages to chat groups of Primary Care Networks (PCNs). By 19 September 2020, 170 clinics across Singapore had taken delivery of booths. All booths and gloves were provided free of charge.

A voluntary and anonymous online survey (Phase 3) was distributed to all GP booth recipients to evaluate their user experience. Structured and open-ended questions enquired about respondent demographics, patterns of booth usage, reasons for booth application, satisfaction with the booth and if the booth influenced their decision to perform swab testing.

Informed consent was taken from study participants and the survey was administered on Qualtrics, with ballot box stuffing disabled. The survey design was supported by Primary Care Research Network, Lee Kong Chian School of Medicine, Nanyang Technological University with ethics approval from NTU (NTU-IRB ref. no. IRB-2020-07-031). Informed consent was also obtained from the subjects of the photo in Fig. 2 for it to be published in an online open-access publication.

Descriptive statistics (i.e., frequencies, percentages, mean, SD) were used to summarize the respondents’ sociodemographics and practice characteristics; Chi-square tests of association was used to examine the association between categorical variables; Pearson’s correlation was used to examine association between continuous variables; independent samples t-test was used to examine differences between groups. All analyses were performed in SPSS for Windows, version 22.0 [19].

## Results

A total of 170 GPs received the swabbing booth and the survey link. 54% of the GPs responded (*n* = 93), of whom 91% (*n* = 85) completed the entire survey. The number of respondents answering each question is indicated in the tables.

### Characteristics of the respondents and their practices

As seen in Table 2, the GPs spanned a wide age range and majority (75%) practiced in public residential estates in small practices (mean = 1.95 doctors).

**Table 2:** Characteristics of respondents (n = 93)

	n (%)	Mean (SD)
**Age**		
30–39	23 (25%)	–
40–49	28 (30%)	–
50–59	35 (38%)	–
60–69	6 (6%)	–
70 and above	1 (1%)	–
**Type of practice**		–
Solo clinic	45 (48%)	–
Group of 2–9 clinics	31 (33%)	–
Group of 10 or more clinics	17 (18%)	–
**Number of doctors in clinic**	–	1.95 (1.20)
**Number of clinic support staff**	–	4.52 (2.68)
**Location of GP clinic**		
public residential estate	70 (75%)	–
shopping mall	13 (14%)	–
private residential estate	4 (5%)	–
office building	5 (5%)	–
industrial estate	1 (1%)	–

### Primary findings on user experience: reasons for booth application and booth satisfaction

The primary findings are summarised in Table 3. The top 3 reasons for applying for the booth were: to increase swabber protection (86%), ease of disinfection (65%) and provided for free (55%).

**Table 3:** Primary findings on user experience

	n(%)
**Reasons for applying for booth (multiple selections accepted)**	**(*n*** **= 92)** ^a^
I felt it would be safer for the swabber	79 (86%)
I felt it would make the disinfection process easier	60 (65%)
It was provided free of charge	51 (55%)
I did not have the necessary equipment to conduct a swab test (e.g. table, privacy screen)	29 (32%)
I felt it would provide privacy for the patients	28 (30%)
Other (reduced patient anxiety, save time without having to wear full PPE, wanted designated work area for swabbing outside clinic)	4 (4%)
**Are you currently using the booth to conduct swab tests?**	**(*n*** **= 86)** ^a^
Yes	68 (79%)
No, I swab without the booth now	16 (19%)
No, I have stopped conducting swab tests in my clinic	2 (2%)
**How likely are you to recommend the booth to another colleague?**	**(n = 85)**^a^
Will strongly recommend	34 (40%)
Will recommend	29 (34%)
Neutral	15 (18%)
Will not recommend	6 (7%)
Strongly will not recommend	1 (1%)
**What do you like about the booth? (multiple selections accepted)**	**(*n*** **= 87)** ^a^
It provides protection to the swabber	80 (92%)
It creates a separate space for swabbing	62 (71%)
It makes the disinfection process easier and quicker	56 (64%)
It is easy to move around	44 (51%)
It is easy to conduct swab tests using the booth	41 (47%)
It provides privacy to the patient	36 (41%)
Others:	2 (2%)
**What do you not like about the booth? (multiple selections accepted)**	**(n = 86)**^a^
Others (e.g. gloves, glove port height)	38 (44%)
Takes up too much space	29 (34%)
Difficult to conduct swab tests using the booth	27 (31%)
Difficult to disinfect	14 (16%)
Troublesome to set up and store	14 (16%)
Inadequate patient privacy	12 (14%)
Inadequate swabber protection	1 (1%)
**Were you swabbing patients prior to receiving the booth?**	**(n = 92)**^a^
Yes	49 (53%)
No	43 (47%)
**How important was getting the booth in your decision to participate in Swab-and-Send-Home (SASH)?**	**(n = 92)**^a^
Very important	32 (35%)
Important	21 (23%)
Somewhat important	23 (25%)
Not important	16 (17%)

^a^ *= number of respondents who answered this question*

We assessed overall satisfaction by whether the GPs were still using the booth at the time of the survey and whether they would recommend it to other GPs. We also assessed satisfaction towards individual attributes of the booth, with questions on likes, dislikes and ergonomics.

### Exploratory analyses of factors associated with user experience

In addition, we performed exploratory analyses (see Table 4) to investigate factors associated with continued use of the booth, ergonomics evaluations, and importance of the booth to participating in swab testing.

**Table 4:** Exploratory analyses of factors associated with user experience

Likes and dislikes correlated with continued booth use			
	Currently swabbing with booth (*n* = 68)	Currently swabbing without booth (*n* = 16)	*p*-value
Likes:			
• It creates a separate space for swabbing	53 (78%)	7 (44%)	0.006^a^
• It is easy to move around	**38 (56%)**	**4 (25%)**	**0.026**
• It is easy to conduct swab tests using the booth	**40 (59%)**	**0 (0%)**	**< 0.001**
• It provides protection for the swabber	65 (96%)	13 (81%)	0.045^a^
• It provides privacy to the patient	**33 (49%)**	**3 (19%)**	**0.030**
• It makes the disinfection process easier and quicker	47 (69%)	7 (44%)	0.057^a^
Dislikes:			
• It takes up too much space	18 (26%)	10 (63%)	0.006^a^
• Difficult to conduct swab tests using the booth	**13 (19%)**	**14 (88%)**	**< 0.001**
• Difficult to disinfect	10 (15%)	4 (25%)	0.320
• Troublesome to set up and store	7 (10%)	7 (44%)	0.001^a^
• Inadequate patient privacy	7 (10%)	4 (25%)	0.117
• Inadequate swabber protection	0 (0%)	1 (6%)	0.038^a^
Commented on gloves	22 (32%)	4 (25%)	0.567
Commented on glove ports	9 (13%)	4 (25%)	0.242
Note. Chi-square tests of associations were performed for all analyses. Results in bold are statistically significant			
^a^ some cells have expected count less than 5			
Comparison of users < 50 years old and ≥ 50 years old			
	< 50 years old (*n* = 50)	≥50 years old (*n* = 42)	p-value
	n (%) or Mean (SD)		
Currently using the booth	36 (72%)	32 (76%)	0.252
Not swabbing prior to receiving booth	18 (36%)	25 (60%)	0.024
Total number of likes	3.45 (1.74)	4.13 (1.34)	0.044
Total number of dislikes	1.47 (1.25)	1.74 (1.33)	0.326
How important was getting the booth to your decision to participate in SASH? (1 = not important; 4 = very important)	2.64 (1.17)	2.88 (1.04)	0.305
How likely are you to recommend the booth? (1 = will strongly recommend; 5 = strongly will not recommend)	2.04 (1.07)	1.85 (0.88)	0.361

Note. Chi-square tests of associations were performed for the analyses involving currently using the booth and not swabbing prior to receiving the booth. Independent samples t-tests were performed for all other analyses

To explore the effect of age on user experience, we also compared responses of users under 50 years old with those 50 years and above. We hypothesized that age influenced users’ attitudes towards the use of swabbing booth as the 50–59 age group supplied the highest percentage of respondents (38%), followed by 40–49 (30%), then 30–39 (25%). 50 years old was chosen as a cutoff as it created two subgroups of similar size. Only 7% of respondents were 60 years and older, possibly because retirement age in Singapore is 62 years old and many GPs may have retired by that age. Thus, we grouped those 60 and over together with the 50–59 year olds for the analysis.

79% of respondents continued using the booth to conduct COVID-19 swabs at the time of filling out the survey. 74% of respondents said that they “would recommend” or “would strongly recommend” the booth to colleagues.

Top three features of the booth which the GPs liked were swabber protection (92%), creation of a separate space for swabbing (71%) and ease of disinfection (64%). We compared those who continued to use the booth to swab and those who were swabbing without the booth (see Table 4). Those who continued to use the booth were more likely to indicate their appreciation for the ease of moving the booth around (56% vs 25%, *p* = 0.026), ease of swabbing (59% vs 0%, *p* < 0.001) and the patient privacy it provided (49% vs 19%, *p* = 0.030). They were less likely to indicate they disliked the difficulty of conducting a swabbing test in the booth (19% vs 88%, p < 0.001).

Top dislikes were that it took up too much space (34%), difficulty in swabbing (31%) and difficulty in disinfecting (16%).

Ergonomics was rated as either poor, adequate or excellent. 56% of the GPs rated the ergonomics of the booth as adequate, 23% excellent and 21% poor. Poorer ratings were correlated with GPs’ heights falling outside the range of 160-180 cm (*p* = 0.033) and the user making free-text comments on the glove ports (*p* = 0.010), but not about the gloves (*p* = 0.630, all Pearson chi-square). Poorer ratings on ergonomics were correlated with selecting the dislike of difficulty in swabbing (*p* = 0.001, Pearson chi-square) and number of total dislikes (*p* < 0.001).

47% of the GPs were not swabbing prior to receiving the booth. 58% of the GPs said the booth was “important” or “very important” to their decision to participate in the SASH programme.

Rating of importance to participation in SASH was correlated with the reason for applying for the booth because the GP did not have the necessary equipment (Pearson correlation *p* = 0.002). It was also correlated with liking the swabber protection (*p* = 0.005), ease of disinfection (*p* = 0.004), ease of conducting swab tests (*p* < 0.001), mobility of the booth (p = 0.002) and patient privacy (*p* = 0.034).

A significantly higher proportion of GPs 50 years and above (60%) were not swabbing before receiving the booth, compared to the younger GPs (36%) (Pearson chi-square *p* = 0.024). Respondents over 50 years tended to indicate more likes than those below 50 (Total number of likes was 4.13 vs 3.45, 2-tailed significance *p* = 0.050).

### Patterns of use

52% of GPs swabbed outdoors or semi-outdoors. 85% of GPs swabbed ≤5 patients a day and 96% of the swabs were done by doctors. Swabbers wore N95 masks (94%), isolation gowns (97%), gloves (96%) and eye protection gear (75%) and 56% changed items of PPE after every swab. Booths were disinfected by clinic assistants (76%) and most often with alcohol (75%). 74% reported that the booth required only one person to move it around.

### Users’ free text comments on booth design

55% of the GPs made free text comments on the booth (see Table 5). Analysis was carried out by two of the authors. Main critiques were on the gloves, height of glove ports and the bulkiness of the booth.

**Table 5:** Themes in free text comments on the booth

Theme	Sub-theme	Examples
**Gloves (35)**	Prefer to swab without long gloves [9]	*“I cut off the hands of the gloves as it was time consuming and difficult to use with the gloves”*
	Wrong size [7]	*“supplied gloves too small. Not used as a result.”*
	Not touch-sensitive enough [6]	*“glooves [*sic*] thick and lac [*sic*] ‘feel’”, “hand gloves are too stiff”*
	Generally hard to use [6]	*“The full length rubber glove that came with booth hard to use”*
	Tear easily [5]	*“Some difficulty applying gloves onto the booth - the gloves tear easily”*
	Hard to insert/remove hands [4]	*“the gloves are too rigid, VERY hard to even get my hands in, granted that I have big hands, size 8*
	Slippery [3]	*“The gloves are slippery and makes handling poor.”*
	Hard to change gloves [1]	*“Gloves that can be easily fitted and changed”*
**Accommodating different-sized users** [20]	Height & restrictiveness of glove port [16]	*“Booth is not user friendly as there is a restriction in terms of height due to the fixed location of the hand glove position. it restricts the height of both the swabber and patients”*
	Could not swab sitting/wheelchair patients [6]	*“it is not wheelchair or elderly friendly.”*
	Cubicle too small or short [2]	*“however, the top of the booth is too low for Caucasian patients”* *“For a ladies [*sic*] frame it’s a good fit but not for the larger built guys”*
**Dimensions** [19]	Too bulky/wanted foldable [10]	*“Try to design a foldable one.”* *“no need to be so bulky and tall”*
	Patient cubicle dimensions [3]	*“Too far for patient. Patient can move away during swab.”*
	Too heavy [3]	*“heavy to push in and out of the clinic after every session”*
**Miscellaneous** [19]	Want it more enclosed [6]	*“There is no “door” to total close patient in so to ensure the aerosol particles are contain within the booth…”*
	Enhance places to put things [5]	*“Put a ledge on patients’ side so the things less likely to drop”* *“compartments to put disinfectants and swabbing materal [*sic*]”*
	Others [8] – 2 or fewer comments per theme	*“Difficult for patient to hear me while swabbing - I bought a mic and speaker set to overcome this”*

Seven respondents expressed appreciation for the booth. One memorable compliment was from an elderly GP:

“*I am very grateful for the booth, without which I would not have started doing the swab. The peace of mind it gives me is tremendous, as I am already 67 and have co morbidity as well. Hence the swab booth gives me the opportunity to help in the fight against Covid. Thank you again.”*

## Discussion

As of 14 November 2020 there were 379 GPs publicly listed as being on the SASH programme [21]. Our study found that 170 GPs had requested a swabbing booth. Around half (47%) of the respondents only started swabbing after receiving the booth, suggesting that the booth helped to increase GP participation in swab testing. Around three-quarters of the users were satisfied with the booth with 79% of the GPs continuing to use it and 74% who would recommend it. Majority of the GPs liked the swabber protection (92%), creation of a separate space for swabbing (71%) and ease of disinfection (64%). 74% of the GPs reported that the booth required only one person to move it, suggesting that our booth design had achieved the objectives of being mobile, lightweight and easy to set up. The survey highlighted some areas of user dissatisfaction: 34% felt the booth took up too much space; 21% rated the ergonomics as poor. Free-text comments often criticized the gloves, height of glove ports and bulkiness of the booth.

### Our swabbing booth in context

During the COVID-19 pandemic, swabbing booths have been developed in countries such as India [20, 22], South Korea [16, 23], the United States [24–26], and Singapore [14] where they have been deployed in settings such as hospitals or testing centres. To our knowledge, ours is the only booth in medical literature designed by GPs for use in GP clinics and deployed on this scale (170 clinics). Ours is the second project to incorporate a user satisfaction survey; one other booth project has reported a 9-question user survey of 8 users.

In terms of form, available swabbing booths can be broadly divided into two types, pressurized and non-pressurized. Pressurized booths [16, 22–25] are sealed cubicles with electrical ventilation and air-cleaning systems. Non-pressurized booths [14, 15, 26] are open-sided structures which are naturally ventilated. Whether pressurized or non-pressurized, both types tend to be large, stationary boxes to contain one standing person. Our booth was an open, non-pressurized, naturally ventilated booth but with unique features not found in other designs: it was compact, designed to be wheeled around by one person, and contained cubicles for swabber and patient which provided some privacy. These were ideas generated by testers during the human-centred design process.

### Human-centred design in a pandemic

We found the human-centred design approach valuable as it generated a product that GPs met the needs of the GPs, increased their participation in swabbing and thus expanded community surveillance for COVID-19. This approach also encouraged innovation and yielded a novel booth design well adapted to the local context.

Due to its iterative nature the human-centred design process can be time-consuming, and even Donald Norman has acknowledged that it can create scheduling problems [27]. However, we were able to progress quickly from design to working product in 2 weeks, and this was partly due to the time saved by having the GP end users co-design the booth while an engineering team rapidly prototyped the design. Other swabbing booths have also been co-designed by clinicians [14, 16, 26], while an alternative approach was design led by engineering teams [20, 22, 24, 25]. In our situation, we found that having GP end users co-design was efficient and direct and resulted in a product that was well-received by GP end users.

### Areas of user dissatisfaction

The survey highlighted certain areas of user dissatisfaction such as booth size, glove port height and gloves. At the design stage, we had already considered these issues but were limited by time, movement and resource constraints during a national lockdown. There was urgency to complete the design in two weeks to be in time for the lifting of the lockdown that we anticipated would increase demand for community swab testing. Under those circumstances, it was difficult to refine the booth further to eliminate user dissatisfaction entirely. There was no immediate solution to making the booth smaller or foldable without compromising the ergonomics or structural integrity. The glove port height was designed for a swabber 1.55–1.75 m tall but the ports were not height adjustable. Although the gloves were the best of several ready-made products available, they still needed improvement to increase dexterity.

Had there been fewer constraints, a more refined booth may have been achieved, perhaps with a choice of sizes and adjustable-height glove ports. It is interesting that in the other booth project which involved a user survey [24], users rated the booth highly on safety (10 ± 0.00 out of a maximum of 10), but rated it most poorly on dexterity of gloves (6.37 ± 2.13 out of 10). This could suggest a need for more research into purpose-built long swabbing gloves.

### Relationship of user satisfaction and user participation in the design process

User satisfaction has been linked to user participation in the design process and user-developer communication [28]. The first 10 pilot GP testers understood the constraints as they had been closely communicating with the design team. Thus, they did not expect a perfect product. However, when the final booth was delivered to 170 practices working independently, it became a “ready-to-use” product. The rest of the 170 GPs were now the consumers, so they may have had higher expectations and therefore expressed more dissatisfaction. On reflection, we recognise that user satisfaction could have been improved by giving attention to creating documentation that communicated the booth features to users and setting up user networks where feedback could be rapidly addressed.

### Learning about booth users

The survey revealed insights on the group of GPs motivated to take up the booth. These were PHPC GPs practicing in small clinics in public residential estates, concerned about swabber protection, disinfection, cost and proper equipment. Despite being supplied PPE, they still requested a swabbing booth, and more than half changed items of PPE after every swab, suggesting an acute consciousness of infection control. Among these GPs, 50 to 59-year-olds constituted the largest group. Those 50 years old and above were significantly less likely to be swabbing before receiving the booth and indicated more likes for the booth. Future studies could survey this group of GPs on their knowledge, attitudes and perceptions towards infection control, and the findings may be used to shape public policy and medical device design.

### Limitations

The 170 GPs represent around one-sixth of the 930 PHPC GPs. The recruited GPs were from established GP networks (e.g. College of Family Physicians Singapore, Primary Care Networks), and their views may not reflect those of PHPC GPs in general. As we did not survey the GPs who did not apply for the booth, we were unable to study the reasons for not applying for the booth and whether this was due to dissatisfaction with the design or due to other reasons. Replicating the study in a different time and place may not produce similar user satisfaction results if other forms of swabber protection were readily available. We also acknowledge that the small sample size and multiple testing may increase the likelihood that significant results may be due to chance.

## Conclusion

The human-centred design approach generated a product that yielded user satisfaction, addressed GPs’ concerns of swabber protection and increased GPs’ participation in swab testing. The booth may be useful in settings where GPs are concerned about swabber protection and space is limited.

## Supplementary Information

**Additional file 1.**

## Data Availability

All data generated or analysed during this study are included in this published article.

## Abbreviations

GP: General Practitioner
HCD: Human-centred design
PHPC: Public Health Preparedness Clinic
PCN: Primary Care Network
SASH: Swab-and-Send-Home
TF: Temasek Foundation
CMC: Camry Medical Centre
ATC: Applied Total Control Treatment Pte Ltd.
