# Correction to: A mobile swabbing booth to address Singapore GPs’ concerns about swabber protection: human-centred design during the COVID-19 pandemic

**DOI:** 10.1186/s12875-021-01565-y

**Published:** 2021-11-12

**Authors:** Boon See Teo, Esther Li, Yi-Lin Khoo, Michelle Evaristo, Yang Fang, Helen E. Smith

**Affiliations:** 1 Camry Medical Centre, 95 Toa Payoh Lorong 4 #01–66, Singapore, 310095 Singapore; 2 Lee Kong Chian School of Medicine, Nanyang Technological University, Experimental Medicine Building, 59 Nanyang Drive, Singapore, 636921 Singapore; 3 Yong Loo Lin School of Medicine, National University of Singapore, NUS Yong Loo Lin School of Medicine NUHS Tower Block, 1E Kent Ridge Road Level 11, Singapore, 119228 Singapore; 4 Duke-NUS Medical School, 8 College Road, Singapore, 169857 Singapore; 5 Temasek International, 60B Orchard Road, #06–18 Tower 2, The Atrium@Orchard, Singapore, 238891 Singapore

**Correction to: BMC Fam Pract 22, 180 (2021)**

10.1186/s12875-021-01531-8

In the original publication of this article [1], the link “10.21979/N9/XRTKJV” was missing under Availability of data and materials section.

## Data Availability

All data generated or analysed during this study are available at 10.21979/N9/XRTKJV. The original article has been corrected.
