# Supplementary material for: A mobile swabbing booth to address Singapore GPs’ concerns about swabber protection: human-centred design during the COVID-19 pandemic
Source: BMC Fam Pract. 2021 Sep 8;22:180. doi: 10.1186/s12875-021-01531-8 (PMC8425014; doi:10.1186/s12875-021-01531-8)
Supplement: Supplementary file 1 — Additional file 1. [file 12875_2021_1531_MOESM1_ESM.docx]

**Supplementary Material**

**Title:**A Mobile Swabbing Booth to Address Singapore GPs’ Concerns About Swabber Protection: Human-Centred Design during the COVID-19 Pandemic

**Authors:**

Boon See TEO, MBBS, MMed(FM), FCFP(S), FAMS^1,2,3,4^; drteobs@gmail.com (Corresponding author)

Esther LI, M(Arch), MFA^1^; philia.projects@gmail.com

KHOO Yi-Lin, BA, MPhil^5^; khooyilin@temasek.com.sg

Michelle MP EVARISTO, BSc, MBA^5^; michelleevaristo@temasek.com.sg

FANG Yang, BBM, PhD^2^; fang.yang@ntu.edu.sg

Helen E SMITH, BMedSci, BMBS, MSc, DM^2^; h.e.smith@ntu.edu.sg

^1^Camry Medical Centre, 95 Toa Payoh Lorong 4 #01-66 Singapore 310095

^2^ Lee Kong Chian School of Medicine, Nanyang Technological University, Experimental Medicine Building, 59 Nanyang Drive, Singapore 636921

^3^ Yong Loo Lin School of Medicine, National University of Singapore, NUS Yong Loo Lin School of Medicine NUHS Tower Block, 1E Kent Ridge Road Level 11, Singapore 119228

^4^ Duke-NUS Medical School, Singapore, 8 College Road, Singapore 169857

^5^Temasek International, 60B Orchard Road, #06-18 Tower 2, The Atrium@Orchard, Singapore 238891

COSMO Slim: Feedback Form for Pilot PHPC Participants

Thank you for providing feedback on your experience using the mobile swab booth. Your feedback is crucial as it will help us further improve the design and functionality of the booth for future users. On behalf of Temasek Foundation, thank you for your efforts in helping Singapore combat COVID-19!

Q1 Age

- Below 29 (1)
- 30-39 (2)
- 40-49 (3)
- 50-59 (4)
- 60-69 (5)
- 70 and above (6)

Q2 Do you work in a solo practice or a group practice?

- Solo (1)
- Group of 2-9 clinics (2)
- Group of 10 or more clinics (3)

Q3 Number of doctors in clinic:

________________________________________________________________

Q4 Number of support staff in clinic:

________________________________________________________________

Q5 Where is your clinic located?

- In an HDB estate (1)
- In a private residential estate (2)
- In a shopping mall (3)
- In an industrial estate (4)
- In an office building (5)

Q6 Which week did you receive the booth?

▼ Week of Mon 25 May (1) ... Week of Mon 31 Aug (15)

Q7 Were you swabbing patients prior to receiving the booth?

- Yes (1)
- No (2)

Q8 Please share the reason(s) for applying for a booth. Choose all that apply:

- I did not have the necessary equipment to conduct a swab test (e.g. table, privacy screen) (1)
- I felt it would be safer for the swabber (2)
- I felt it would provide privacy for the patients (3)
- I felt it would make the disinfection process easier (4)
- It was provided free of charge (5)
- Other: (6) ________________________________________________

Q9 How important was getting the booth in your decision to participate in Swab-and-Send-Home (SASH)?

- Not important (1)
- Somewhat important (2)
- Important (3)
- Very important (4)

Q10 On average, how many patients do you swab a day?

- <3 patients (1)
- 3-5 patients (2)
- >5 patients (3)

Q11 Do you perform swab tests indoors or outdoors?

- Indoors (1)
- Outdoors (no shelter) (2)
- Semi-outdoors (with shelter) (3)

Q12 How many people are required to move the booth around the clinic?

- 1 person (1)
- 2 persons (2)
- 3 persons (3)

Q13 Who uses the booth to conduct the swab tests?

- Clinic assistant (1)
- Doctor (2)

Q14 What PPE does the swabber wear? (Choose all that apply)

- Eye protection gear (1)
- N95 mask (2)
- Surgical mask (3)
- Isolation gown (4)
- Gloves (5)
- No PPE (6)

Display This Question:

If 14. What PPE does the swabber wear? (Choose all that apply) != No PPE

Q15 How often does the swabber change PPE? (Choose all that apply)

- After every swab (1)
- After a series of consecutive swabs (2)
- At the end of the clinic session (3)

Display This Question:

If 14. What PPE does the swabber wear? (Choose all that apply) != No PPE

Q16 16. Which PPE does the swabber change? (Choose all that apply)

14. What PPE does the swabber wear? (Choose all that apply) = Eye protection gear

- Eye protection gear (1)

14. What PPE does the swabber wear? (Choose all that apply) = N95 mask

- N95 mask (2)

14. What PPE does the swabber wear? (Choose all that apply) = Surgical mask

- Surgical mask (3)

14. What PPE does the swabber wear? (Choose all that apply) = Isolation gown

- Isolation gown (4)

14. What PPE does the swabber wear? (Choose all that apply) = Gloves

- Gloves (5)

Q17 Who disinfects the booth?

- Clinic assistant (1)
- Doctor (2)

Q18 What do you use to disinfect the booth? (Choose all that apply)

- Alcohol/ethanol (1)
- BKC (benzalkonium chloride) (2)
- Dettol (3)
- Soap and water (4)
- Bleach (5)
- UVC (6)
- Sunlight (7)
- Others: (8) ________________________________________________

Q19 How do you find the size of the booth?

- Too bulky (1)
- Just nice (2)
- Too small (3)

Q20 How do you find the ergonomics of the booth?

- Excellent (1)
- Adequate (2)
- Poor (please share why) (3) ________________________________________________

Q21 Which size of long gloves are you using on the booth?

- Free “F” size (1)
- Large “L” size (2)
- I bought my own (3)
- I don’t know (4)

Q22 How is the fit of the gloves?

- Too big (1)
- Just right (2)
- Too small (3)

Q23 If you are comfortable, please share your height.

- <160 cm (1)
- 160-169cm (2)
- 170-180cm (3)
- >180cm (4)

Q24 Are you currently using the booth to conduct swab tests?

- Yes (1)
- No, I swab without the booth now (2)
- No, I have stopped conducting swab tests in my clinic (3)

Q25 What do you like about the booth? Choose all that apply:

- It creates a separate space for swabbing (1)
- It is easy to move around (2)
- It is easy to conduct swab tests using the booth (3)
- It provides protection to the swabber (4)
- It provides privacy to the patient (5)
- It makes the disinfection process easier and quicker (6)
- Others: (7) ________________________________________________

Q26 What do you not like about the booth? Choose all that apply:

- Takes up too much space (1)
- Troublesome to set up and store (2)
- Difficult to conduct swab tests using the booth (3)
- Inadequate swabber protection (4)
- Inadequate patient privacy (5)
- Difficult to disinfect (6)
- Others: (7) ________________________________________________

Q27 How likely are you to recommend the booth to another colleague?

- Will strongly recommend (1)
- Will recommend (2)
- Neutral (3)
- Will not recommend (4)
- Strongly will not recommend (5)

Q28 Do you have any other suggestions on ways to improve the booth?

________________________________________________________________

________________________________________________________________

________________________________________________________________

________________________________________________________________

________________________________________________________________

Q29 Please share any other feedback you have.

________________________________________________________________

________________________________________________________________

________________________________________________________________

________________________________________________________________

________________________________________________________________
